# *Candida albicans* Sap6 Initiates Oral Mucosal Inflammation *via* the Protease Activated Receptor PAR2

**DOI:** 10.3389/fimmu.2022.912748

**Published:** 2022-06-29

**Authors:** Rohitashw Kumar, Isolde Gina Rojas, Mira Edgerton

**Affiliations:** Department of Oral Biology, University at Buffalo, Buffalo, NY, United States

**Keywords:** oral epithelial cells, *Candida albicans*, secreted aspartyl protease (Sap6), protease activated receptor 2, cytokines and chemokines, epithelial barrier break, p38 MAKP signaling

## Abstract

*Candida albicans* Sap6, a secreted aspartyl protease (Sap), contributes to fungal virulence in oral candidiasis. Beside its protease activity, Sap6 contains RGD (RGDRGD) motif required for its binding to host integrins. Sap6 activates immune cells to induce proinflammatory cytokines, although its ability to interact and activate human oral epithelial cells (OECs) remain unknown. Addition of purified recombinant Sap6 (rSap6) to OECs resulted in production of IL-1β and IL-8 cytokines similar to live hyphal *C. albicans*. OECs exposed to rSap6 showed phosphorylation of p38 and MKP1 and expression of c-Fos not found with *C. albicans Δsap6*, heat-inactivated Sap6, or rSap6_Δ*RGD*_. Heat inactivated rSap6 was able to induce IL-1β but not IL-8 in OECs, while rSap6_Δ*RGD*_ induced IL-8 but not IL-1β suggesting parallel signaling pathways. *C. albicans* hyphae increased surface expression of Protease Activated Receptors *PAR1*, *PAR2* and *PAR3*, while rSap6 increased *PAR2* expression exclusively. Pretreatment of OECs with a PAR2 antagonist blocked rSap6-induced p38 MAPK signaling and IL-8 release, while rSap6_Δ*RGD*_ had reduced MKP1 signaling and IL-1β release independent from PAR2. OECs exposed to rSap6 exhibited loss of barrier function as measured by TEER and reduction in levels of E-cadherin and occludin junctional proteins that was prevented by pretreating OECs with a PAR2 antagonist. OECs treated with PAR2 antagonist also showed reduced rSap6-mediated invasion by *C. albicans* cells. Thus, Sap6 may initiate OEC responses mediated both through protease activation of PAR2 and by its RGD domain. This novel role of PAR2 suggests new drug targets to block *C. albicans* oral infection.

## Introduction

*Candida albicans* causes both localized and disseminated systemic infections, particularly in immunocompromised patients and individuals living with chronic diseases (1). However, the vast majority of host-fungal interactions are benign in that *C. albicans* is a component of healthy oral and gut mycobiome (2). *C. albicans* virulence is correlated with hyphal formation and secretion of hyphal-specific proteins such as candidalysin that elicit cytokine release from oral epithelial cells (OEC) and cause epithelial cell damage (3). Recent work by Witchley et al. (4) showed that *C. albicans* hyphae formation promoted virulence in the mammalian gut that was dependent upon expression of hyphal-specific virulence factors. Using an *in vivo* fitness screen of 52-hyphal-associated genes, they found that *C. albicans* Sap6 (secreted aspartyl protease) conferred the highest inhibition of gut commensalism, and proposed a new paradigm that levels of hyphal specific products (primarily Sap6) trigger an immune response when a threshold level is exceeded. Similarly, candidalysin caused release of cytokines including IL-1β and IL-6 in epithelial cells and signaled synergistically with IL-17 to increase cytokine production (5), thus allowing the host to discriminate between damaging and non-damaging hyphae (5–7). Sap6 and closely related secreted Sap5 are also inflammasome activators and contribute to immunopathogenesis in vulvovaginal candidiasis (8), however little is known about their immunogenic role in oral candidiasis. Sap6 is a potent inducer of IL-1β and IL-18 production in monocyte-derived macrophages and dendritic cells (9), however whether *C. albicans* secreted Sap6 itself can activate immune responses in oral epithelial cells remains unclear.

The *C. albicans* genome encodes for 10 related *SAP* family members that have long been known to have a role in fungal pathogenesis. All Sap family members are aspartyl proteases with a catalytic cleft region, although each Sap has varying substrate cleavage site specificities (10). Among these 10 *SAP* gene products, Saps1-3 (yeast associated) and Sap4-6 (hyphal associated) are closely related subfamilies. Sap2 is the most abundant protease expressed by the yeast form *C. albicans*, and Saps1-3 have an functional role in nutrient acquisition and induction of host immune responses (11). Saps4-6 have been associated with tissue invasion and damage accompanying their high levels of hyphal-specific expression and release in the host environment (11). *SAP6* and *SAP5* were the most highly expressed genes in fungal cells recovered from infected mouse tongues during oral candidiasis (12), and we found that over-expression of *SAP6* resulted in hyper-virulence in murine oral candidiasis (13). Sap6 and Sap5 are structurally redundant proteases but have different substrate specificity and functions (10, 14). Both Sap5 and Sap6 have RGDRGD integrin-binding motifs that allow integrin-mediated binding, endocytosis and caspase activation in epithelial cell lines and human platelets (15). Although Sap6 is involved in epithelial binding and invasion (13, 16), little is known about how its protease functions might mediate oral epithelial cell pro-inflammatory responses.

Protease-Activated Receptors (PARs) are surface localized G-protein-coupled receptors that have a unique mechanism of activation initiated by host or microbial secreted proteases. PARs are a family of four receptor proteins each activated by different proteases PAR1 and PAR2 are highly expressed in OECs and protease activity further increases their expression levels. PAR1 is cleaved and activated by thrombin and PAR2 by trypsin, resulting in phosphorylation of MAPKinase, ERK1/2, p38 and the transcription factor AP-1 (17–19). Oral bacteria *Porphyromonas gingivalis* or *Aggregatibacter actinomycetemcomitans* secretes cysteine proteases that activate PAR2 in OECs, and these proteases have been implicated as inflammatory mediators of periodontitis (15, 20, 21). Therefore, we expected that fungal proteases such as Sap6 may also activate epithelial PAR2 resulting in secretion of inflammatory cytokines.

Activation of PAR2 leads to downstream signaling events that not only mediate inflammation, but also degradation of OEC junction proteins and increase cell barrier permeability in a p38 MAPK dependent manner (22, 23). Both bacterial and fungal secreted proteases are able to disrupt mucosal barriers by degrading junction proteins including E-cadherin, resulting in epithelial permeability (24–27). *C*. *albican*s hyphae activate MKP1 phosphorylation and c-Fos activation leading to cytokine release and epithelial damage (28, 29) However, it is not known whether epithelial PAR2 participates in this signaling cascade as a receptor for hyphal secreted Sap6. Despite the well-known association between Sap6 and fungal virulence, its contribution towards hyphal-driven epithelial activation has remained unclear.

We hypothesized that *C. albicans* aspartyl proteases are involved in activating epithelial immune responses, and are responsible for fungal virulence in the context of oral candidiasis. Here, we demonstrate that *C. albicans* Sap6 specifically induces IL-1β and IL-8 secretion in primary OECs that is mediated by MAPK p38 and c-Fos activation. We show for the first time that Sap6 activated OEC PAR2 signaling through p38 phosphorylation and IL-8 release while its RGD domain was needed for IL-1β release apart from PAR2. Furthermore, PAR2 activation by Sap6 resulted in increased hyphal invasion and damage of the epithelial barrier. Thus, PAR2 signaling is a novel and unexplored arm of oral epithelial immunity that comprises one mechanism for threshold sensing of fungal pathogens.

## Methods

### Strains and Growth Conditions

*C. albicans* strains used in the manuscript is listed **supplement Table 1**. The strains of *C. albicans* were regularly maintained on YPD agar (Difco, Detroit, MI). For experiments, a single colony of *C. albicans* was inoculated into 10 ml of YPD medium and incubated at 30°C at 220 rpm for 20 h. Overnight grown *C. albicans* cultures were washed twice in 1X phosphate buffer saline (PBS) and cell density was adjusted using a hemocytometer.

### Chemicals

The peptide SLIGKV (PAR2-AP), FSLLRY(PAR2-ANT) and VKGILS (PAR2 AP control peptide) were purchased from Tocris Biotechne. The MAPK p38 inhibitor SB203580 was purchased from Santacruz and was prepared in ethanol as directed by the manufacturer. RGD peptide (Sigma Aldrich) was dissolved in deionized water as directed by manufacturer.

### Recombinant Secreted Aspartyl Protease (Saps) Purification

*Pichia pastoris* strains containing secretory proteins Sap5, Sap6 and Sap6_ΔRGD_ were kindly provided by Dr. Michel Monod (CHUV, Lausanne, Switzerland) and Dr. Jordan Tang (OMRF, Oklahoma City, USA). These secretory proteins Sap5, Sap6 and Sap6_ΔRGD_ were overexpressed in *P. pastoris* and secreted into the growth medium. The supernatants were used for protein purification as described earlier with some modifications (14, 30). *P. pastoris* strain containing recombinant protein was grown in 1000 mL of buffered complex glycerol medium (BMGY) (0.1 M potassium phosphate buffer at pH 6.0, containing 1% yeast extract, 2% peptone, 1.34% YNB without amino acids, 1% (w/v) glycerol, and 4 x 10^-5%^ biotin) and grown in a shaking incubator(220 rpm) at 30°C to OD_600_ = 6-10. Cells were harvested and resuspended in 200 mL of BMMY medium containing 0.5% (w/v) methanol and were incubated for 48 h. The supernatant containing recombinant protein was collected by centrifugation, filter sterilized (0.2 µm), concentrated and dialyzed against a 100-fold volume of 10 mM sodium citrate buffer (pH = 5.0). Recombinant Sap (rSap6, rSap5 or rSap6_ΔRGD_) from were first purified using Macro-prep High S column (Bio Rad, USA), eluted with a gradient of 10 to 200 mM sodium citrate buffer (pH=5.0). The fractions containing recombinant proteins were pooled, concentrated and purified using Sephadex G-100 column (Pharmacia, Piscataway, NJ, USA) eluted with a 10mM sodium phosphate buffer (pH 7.0). Recombinant proteins (rSap5, rSap6, and rSap6_ΔRGD_) were further concentrated and quantified using BCA protein assay (Thermo Scientific), Purified recombinant Sap6 was heat-inactivated by autoclaving at 120°C for 20 min.

### Epithelial Cell Lines

Human primary oral epithelial cells (OECs) were collected from gingival tissues of healthy adults (kindly provided by Dr. Ozlem Yilmaz, MUSC, Charleston, SC). OECs were cultured as monolayers in serum-free keratinocyte growth medium (KGM™-2 Keratinocyte Growth Medium-2 GIBCO) at 37°C in 5% CO_2_ and used for experimentation at ∼90% confluence. TR146 buccal epithelial carcinoma cell line (ECACC) monolayers were grown in Dulbecco’s Modified Eagle’s Medium-F12 (DMEM-F12) supplemented with 10% fetal bovine serum (FBS) to 90% confluence and experiments were carried out in serum-free DMEM/F12 medium. Both primary OECs and TR146 cells were used following 4-10 passages for experiments.

### Epithelial Cell Activation

To stimulate and activate, confluent OECs were grown in 12-well plates (Corning) using KGM-2 medium, then *C. albicans* (1x10^6^ cells/ml), or rSap6 (10 μM), or rSap6 (Inact), or rSap6 _ΔRGD_ were added and incubated for 3 h (for cell lysates) or 24 h (culture supernatants) at 37°C with 5% CO_2_. For blocking experiments, OECs were pre-incubated with PAR2-AP (100 μM), PAR2-ANT (100μM), RGD (10μM) or an anti-integrin antibody (anti-integrin αM CBR1/5, Santa Cruz Biotech) in a 1:100 dilution for 24 h prior to addition of rSap6 (10µM).

### Protein Isolation and Western Blotting

Total cell lysates from OECs treated as per different experimental conditions were isolated as described (31). For OECs lysates, 400 µl RIPA buffer (Santa Cruz Biotech) were added to each well of 12 well plate, incubated at 4°C for 30 min with gentle shaking. The OECs cell lysates were transferred to microfuge tubes, and centrifuged for 10 min at 21,000 x g at 4°C to remove cellular debris, quantified using BCA protein assay and stored at -80°C till further use. For immunoblotting, OECs cell lysate (20µg) were loaded on 12% or 15% SDS- PAGE and used for western blotting using PVDF membrane (BioRad). After transfer, PVDF membranes were blocked in 5% non-fat milk or BSA (Sigma) in 1X Tris Buffered Saline with 0.1% Tween-20 TBST, pH 7.2) at room temperature for 1 h followed by probing with primary antibody (diluted in 5% BSA) for 16 h at 4°C. Next, the membranes were washed twice with TBST and probed with a secondary antibody (diluted in 5% non-fat milk) for 1 h at room temperature. The signals were detected using SuperSignal West Pico detection kit (Thermo Scientific) according to manufacturer’s instructions. Density of protein bands was quantified using Image Lab 6.0 (BioRad), and normalized using a loading control. Relative fold differences were calculated as ratio of normalized band intensity of experimental and control samples. Phospho-DUSP1/MKP1 (Ser359) rabbit mAb (2857), Phospho-p38 MAPK (Thr180/Tyr182) rabbit mAb (9211), cleaved-IL-1β (Asp116) (D3A3Z) rabbit mAb (83186), and c-Fos rabbit mAb (2250) antibodies were purchased from Cell Signaling Technologies. E-cadherin mouse mAb (G-10) (sc-8426), Occludin mAb (sc-133256) and IL-8 mouse mAb (sc-376750) were purchased from Santacruz. The secondary antibodies anti-rabbit IgG (Cell Signaling Technology) and anti-mouse m-IgGκ BP-HRP: (sc-516102, Santa Cruz) were used at 1:4000 dilution

### Cytokine Array and ELISA

For detection of cytokines/chemokines released in cell supernatant, OECs were stimulation for 24 h under various experimental conditions. For arrays, Proteome Profiler Human Cytokine Array B (R&D Systems) membranes were incubated for 12 h with OEC supernatant (400 μl) at 4°C, washed extensively with array buffer supplied in the kit, then incubated with biotinylated antibody cocktail for 1 h. Streptavidin-HRP was added for 30 min followed by chemiluminescent substrate reagent (Bio-Rad). Chemiluminescence was documented using a ChemiDoc MP imaging system (Bio-Rad). For ELISA culture supernatant were diluted to 1:100 in assay buffer and IL-8 or IL-1β were quantified using human IL-8 ELISA MAX™ or IL-1β ELISA MAX™ (BioLegend Inc, USA) as per manufacturer’s instructions.

### Immunofluorescence of Oral Epithelial Cells

Confluent OECs grown on coverslips were treated with rSap6, rSap6 (Inact), PAR2-AP and PAR2-ANT for 2 or 24 h. After treatment, cells were then fixed with 4% para-formaldehyde in PBS for 20 min at 20°C and washed three times for 5 min with PBS. Fixed cells were blocked with 1% BSA for 1 h, then incubated with primary antibodies for Par2 (Cell Signaling) or E-cadherin (Santa Cruz) at 1:1000 for 12 h at 4°C. Cells were again washed three times with PBS before addition of the secondary antibody (anti-rabbit IgG Alexa Fluor^®^ 488; Abcam) for 2 h at room temperature. Cells were stained with Hoechst (Sigma) to stain nuclei, washed, and cover glass slips were mounted on slides (Globe Scientific Inc.) using fluorescent mounting media (Dako). Cells were imaged using a Zeiss Axio Observer Z1 inverted fluorescent microscope (Carl Zeiss, Germany). The images were processed using ImageJ software.

### Epithelial Invasion Assay

OECs or TR146 monolayers were cultured on 15 mm glass round coverslips (VWR Vista vision) in 12 well culture plates at 37°C with 5% CO_2_ to confluence. For invasion, *C. albicans* (1x10^6^ cells/ml) were added to each well at 37°C with 5% CO_2_ for 4 h. Then, epithelial monolayers were washed three times with PBS to remove non-adherent *Candida* cells, and fixed with 4% formaldehyde for 15 min. Non-invasive fungal cells were stained using rabbit anti-*C. albicans* antibody (Origene 1:1000 in PBS) at 4°C for 16 h, followed by secondary goat anti-rabbit-Alexa Fluor 488 antibody (Abcam) 1:2000 in PBS) for 1 h. After staining, epithelial monolayer cells were permeabilized using 0.1% Triton X-100 for 20 min at 37°C in the dark. After permeabilization, total *C. albicans* cells were stained with 1mg/ml Calcofluor White (sigma) for 20 min. Coverslips were rinsed in water and mounted on slides using 1-2 drops of fluorescent mounting medium (Dako) and allowed to air-dry for 1-2 h in the dark. Fluorescence images were documented using a Zeiss Axio Observer Z.1 microscope and processed by ImageJ software. The number of invading *C. albicans* cells were determined as percentage of number of invading hyphae divided by the total number of Candida cells. At least 200 C*. albicans* cells in different fields were counted to calculate percentage invasion. For various experiments, epithelial monolayers were pre-incubated with PAR2-AP (100 μM), PAR2-ANT (100 μM), rSap6 (10 μM) or RGD (5 μM) peptide for 1 h before addition of *C. albicans* (1x10^6^ cells/ml).

### RNA Isolation and Real Time PCR

For total RNA isolation from OECs, TRIzol (1 ml) was added to individual wells of 12-well plate to lyse OECs mechanically. The lysed OECs were transferred to 2 ml microfuge tubes, and incubated at room temperature for 5 min. Next, chloroform (200 µl for 1ml of TRIzol) was added and vortexed for 15 sec, then centrifuged at 21,000 x g for 10 min at 4°C. The upper clear aqueous layer was removed carefully to another microfuge tube and mixed with 100% ethanol to precipitate total RNA from OECs. Total RNA was further purified using RNAeasy kit (Qiagen), quantified and used to quantitate gene expression of the following genes (*IL-1β*, *IL-8*, *PAR1*, *PAR2*, *PAR3* and *PAR4*). For qPCR analysis, total cDNA was synthesized using 1 μg of RNA in a 20 μl reaction mixture by iScript cDNA synthesis kit (Bio-Rad). All samples were prepared with SsoAdvancedTM Universal SYBR^®^ Green Supermix (Bio-Rad), with 1μl of cDNA template in 20 μl reactions and 150 nM of forward and reverse primers. The sequences of primers used were: *IL-8 qRT*Forward : CACTGCGCCAACACAGAAAT;*IL8*qRTReverse;GCTTGAAGTTTCACTGGCATC; *IL1βqRT*Forward;AAGCAGCCATGGCAGAAGTA; *IL-1βqRT*Reverse; GGTGGTCGGAGATTCGTAGC *PAR1qRT*Forward;CTTTTCCGGCAGTGATTGGC *PAR1qRT*Reverse;GGGACTGCATGGGATACACC; *PAR2qRT*Forward CTGACTTTCTCTCGGTGCGT *PAR2qRT*Reverse;GTTCCTTGGATGGTGCCACT *PAR3qRT*Forward; GCTTTGTGCCTGGGTAGTCT *PAR3RT*Reverse GGAGCTCCTTGCACTATGC *PAR4qRT*Forward;ATGACAGCACGCCCTCAATC *PAR4qRT*ReverseACCAGGACCAGCCCATAGAG; *GAPDHqRT*Forward GACAGTCAGCCGCATCTTCT *GAPDHqRT*Reverse GCGCCCAATACGACCAAATC. For real time PCR, samples were denatured for 3 min at 95°C, then PCR reactions were cycled 40 times using the following parameters: 95°C for 30 s, 50°C for 30 s, and 72°C for 30 s using a CFX Connect™ Real time system (Bio-Rad). Each reaction set up contained negative RNA controls, no-template controls, and positive genomic DNA controls. The quantification cycle (Cq) value was calculated using CFX Maestro software. Relative fold changes in gene expression of genes of interest were calculated compared to GAPDH that is not regulated in our experimental setting.

### Measurement of TEER in OECs

OECs were grown on 3μm trans-well inserts in 24 well plates (Corning) to confluence before addition of rSap6 at different concentrations. Transepithelial electric resistance (TEER) was measured over 48 h after addition of Sap6 using an EVOM2 epithelial volt ohmmeter (World Precision Instruments, Germany). All measurements were performed on a minimum of triplicate wells.

### Statistical Analysis

Experimental data was analyzed using GraphPad Prism version 9 (GraphPad Software, San Diego, CA, USA). All experiments were repeated at least three independent times to ensure reproducibility, and data from a representative experiment are shown as mean ± SEM. Unpaired t-tests, one-way or two-way ANOVA were used to determine statistical significance.

## Results

### Sap6 Induces IL-8 and IL-1β Production in OECs

**Figure 1:**
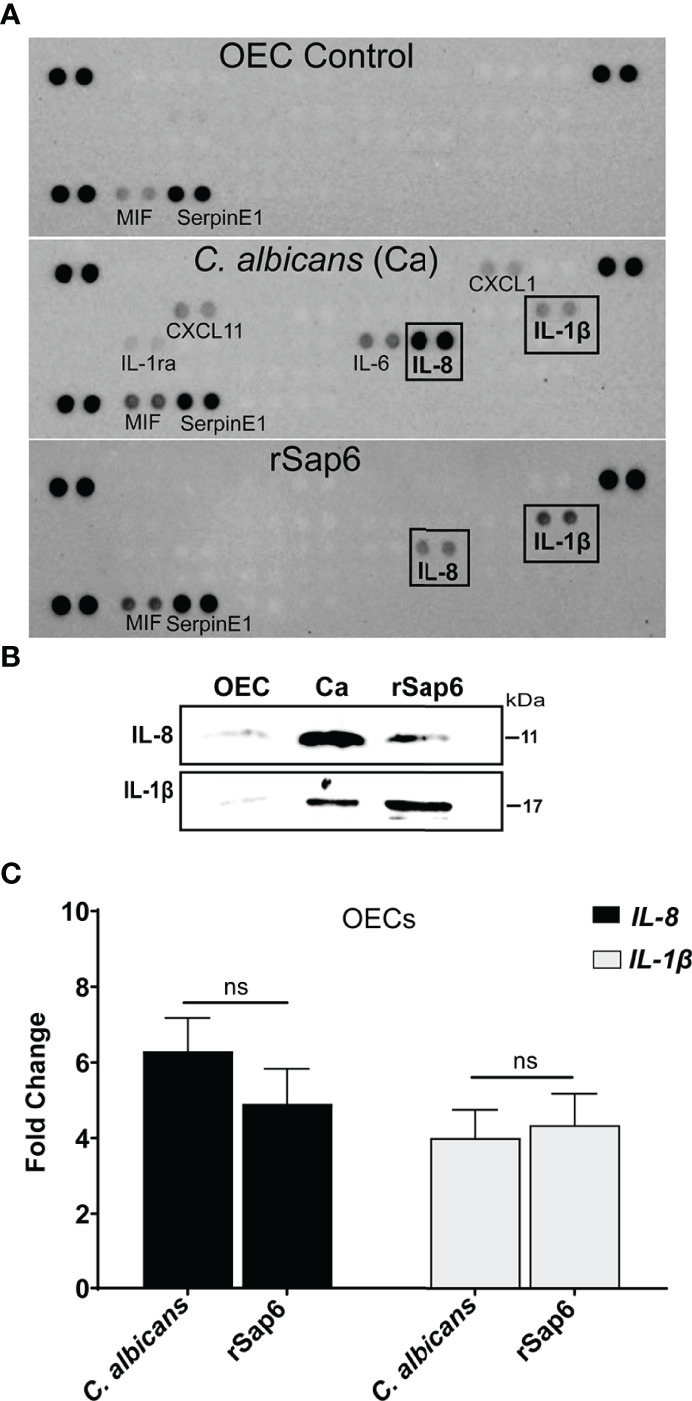
Secreted aspartyl protease Sap6 induces IL-1β and IL-8 in primary oral epithelial cells. **(A)**. Cytokine profiling of OEC supernatant treated with *Candida albicans* (Ca) or recombinant Sap6 (rSap6) was examined using a proteome profiler Cytokine Array Panel **(A)** Proinflammatory IL-1β and IL-8 cytokines were detected in both Ca or Sap6 treated OECs although Ca induced release of additional cytokines in OECs. **(B)** OEC culture supernatants were collected following treatment with Sap6 or Ca for 24 h, then immunoblotted to confirmed secretion of IL-8 and IL-1β. **(C)** Total RNA was isolated from OECs incubated with either Ca or Sap6 for 24 h, then expression levels of *IL-8* and *IL-1β* quantitated using RTqPCR using Il-8 and IL-1β specific primers with GAPDH as a housekeeping gene. There was no significant difference in expression levels of *IL-8* or *IL-1β* between OECs treated with Ca or Sap6. Experiments were performed at three independent time points in triplicate; and the mean ± SEM were analyzed using one-way ANOVA. ns, non significant.

We investigated first whether cytokine release occurs in OECs following exposure to Sap6 protein compared with *C. albicans* cells. OECs were grown to confluence and stimulated with either recombinant purified Sap6 (rSap6, 10 μM) or *C. albicans* SC5314 for 24 h at 37°C. Supernatants were collected, centrifuged to remove cells, then used for cytokine array analysis (**Figure 1**). Similar to previous studies (28, 32–35), incubation of OECs with *C. albicans* cells induced prominent release of IL-8, as well as proinflammatory cytokines IL-1β, IL-1ra, IL-6, CXCL11, and CXCL1 (**Figure 1A**). Incubation with purified rSap6 alone also stimulated OEC secretion of IL-1β and IL-8, while other cytokines were not detectable at this concentration of added Sap6. Release of IL-8 and IL-1β in OEC supernatants by either rSap6 or *C. albicans* cells were confirmed by Western blotting (**Figure 1B**). Total RNA from rSap6 and *C. albicans* treated OECs was isolated and expression levels of IL-8 and IL-1β was examined by RTqPCR (**Figure 1C**). The increase in expression levels for genes encoding IL-8 or IL-1β in OECs treated with rSap6 (10 μM) were not statistically different than OECs exposed to *C. albicans* cells. Thus, purified rSap6 elicited OECs production of a narrower range of cytokines including IL-8 and IL-1β compared with *C. albicans* cells at the tested concentration, but their levels of production were comparable to that induced by *C. albicans*.

### Sap6 Initiated Activation of MAPK Signaling in Primary OECs

**Figure 2:**
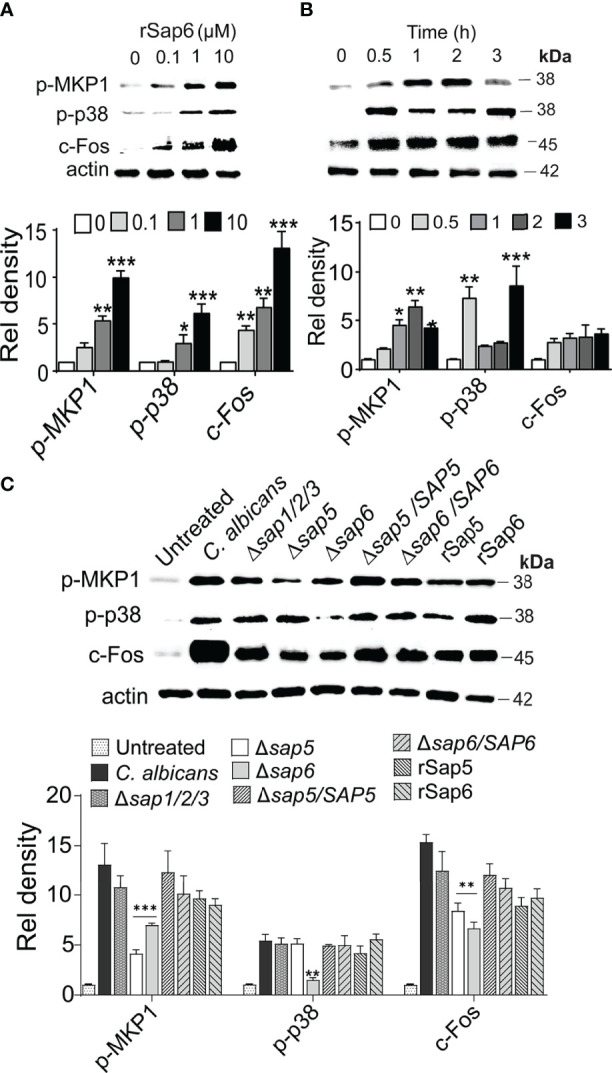
rSap6 activates p-38, MKP1, and c-Fos MAPK signaling pathway **(A)** OECs were treated with rSap6 (0-10µM) for 3 h, then OEC cell lysates (20 µg) were immunoblotted using p-p38, p-MPK1 and c-Fos antibodies. rSap6 showed concentration dependent significant increase in MAPK phosphorylation and c-Fos activation, with 10µM rSap6 showing highest activation of OECs. **(B)** OECs were treated with rSap6 (10µM) for 0.5 - 3 h and cell lysates were immunoblotted. MKP1 phosphorylation gradually increased up to 2 h, then decreased at 3 h while p38 phosphorylation had an inverse response suggesting feedback between MKP1 and p38. **(C)** *Candida albicans* WT (SC5314) cells and *SAP* deletion strains along rSap5 (10µM) and rSap6 (10µM) were incubated with OECs for 3 h. There was no change in p38/MKP1 MAPK signaling in *Δsap1/2/3* compared to WT cells, while Ca*Δsap5* or Ca*Δsap6* resulted in reduced p38/MKP1 phosphorylation and c-Fos expression that were restored in OECs incubated with complemented strains (*Δsap6/SAP6* and *Δsap5/SAP5*) and were comparable with rSap6 and rSap5 proteins. β actin was used as a loading control. Western blots were analyzed using ImageLab software for relative density and significance determined using two-way ANOVA. P values are p ≤ 0.5 (*), p ≤ 0.01 (**), p ≤ 0.001 (***) and can be removed. Immunoblot data are representative of three independent experiments.

Since IL-1β and IL-8 signaling is downstream result of MKP1/p38/c-FOS signaling in OECs upon hyphal invasion, we asked whether Sap6 protein also could initiate such signaling. Indeed, addition of rSap6 to OECS resulted in phosphorylation of both MKP1 and p38 and increased expression of c-Fos transcription factor (TF) in a concentration dependent manner (**Figure 2A**). Densitometry analysis showed a significant increase in MAPK MKP1, p-38 phosphorylation and c-Fos levels when cells were stimulated with either 1 µM or 10 µM Sap6. We selected a concentration of rSap6 (10 μM) for further experiments based upon this result as well as the cytokine array (**Figure 1A**). During *C. albicans* hyphal invasion, OEC MAPK responses have been characterized by initial p38 phosphorylation followed by phosphorylation of MPK1 to provide negative feedback regulation of this response. We found similar signaling cascade in OECs following rSap6 exposure so that p38 phosphorylation was the most robust signal detected within 30 min. Densitometry analysis of the protein bands showed that p38 levels were significantly higher at 30 min and at 3 h, whereas there was gradual and significant increase in Mkp1 levels up to 2 h (**Figure 2B**). As expected for their activator/repressor roles, we observed that levels of p38 and MKP1 phosphorylation were inversely related over time of rSap6 exposure (**Figure 2B**). Next, we asked whether other Sap family member proteins might have a role in OEC signaling by examining *C. albicans SAP* gene deletion mutants (**Figure 2C**). We compared live *C. albicans* hyphal cells with a triple deletion mutant of Sap1, Sap 2 and Sap 3 (*Δsap1/2/3*) with parental WT cells and found no significant reduction in MAPK signaling, suggesting that this group of co-expressed Saps are not involved in OEC responses. In contrast, incubation of OECs with fungal cells carrying deletions of *SAP5* or *SAP6* resulted in significantly reduced Mkp1 or p38 phosphorylation and c-Fos expression compared with purified rSap6 and rSap5 and with WT cells. OEC responses were restored by incubation with each respective complemented strain (*Δsap6/SAP6* and *Δsap5/SAP5*) and were comparable to treatment of OECs with purified Sap5 and Sap 6 proteins (**Figure 2C**). Thus, both related Sap5 and Sap6 are able to induce MAPK (both Mkp1 and p38) signaling in OECs, while Saps1-3 were not able to induce this signaling response.

### Sap6 Mediated MAPK Activation and IL-8 and IL-1β Release Depend on Its Protease Activity and RGD Motif

**Figure 3:**
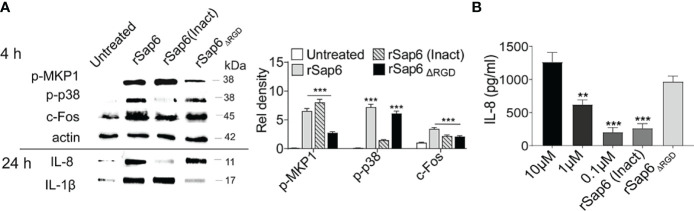
Both Sap6 protease activity and its RGD motif modulate MAPK signaling and cytokine release. OECs were treated with rSap6, heat inactivated rSap6 (Inact) and rSap6_Δ_ *_RGD_* for 3 h (for cell lysates) or 24 h (for culture supernatants). **(A)** OEC cell lysates treated with rSap6 (Inact) failed to induce p38 phosphorylation and IL-8 release without affecting MKP1 phosphorylation, c-Fos expression or IL-1β compared to active rSap6. OECs treated with rSap6*ΔRGD* showed reduced MKP1 phosphorylation and IL-1β release without affecting p38 phosphorylation, c-Fos and IL-8 levels. **(B)** OEC culture supernatant was collected after 24 h of treatment with rSap6 (0.1-10µM), rSap6 (Inact, 10µM) or rSap6*ΔRGD* (10µM) and IL-8 was quantitated by ELISA. IL-8 secretion was dose dependent with rSap6(0.1-10µM) and was significantly (P<0.001) reduced by treatment with rSap6 (Inact) compared with active rSap6 or rSap6*ΔRGD*. Data are mean ± SEM and are representative of three independent experiments. Significance was calculated using two-way ANOVA compared to untreated OECs. P values are, p ≤ 0.01 (**), p ≤ 0.001 (***).

Since both protease activity and the RGD integrin-binding domain within Sap6 are important components in *C. albicans* Sap6-mediated pathogenesis (14), we tested the requirement of either protease activity or the RGD domain for epithelial MAPK activation and cytokine release. OECs were incubated with rSap6 (10μM), heat inactivated (rSap6 Inact) rSap6 (10 μM) and a Sap6 protein missing its RGD domain (rSap6_Δ_ *_RGD_*; 10 μM) over 4 h and 24 h. OEC lysates and culture supernatants were collected for Western blotting to detect MAPK activation (4 h) and IL-8 and IL-1β secretion (24 h) respectively (**Figure 3A**). Densitometry analysis showed that OECs treated with rSap6 (Inact) showed reduced p38 phosphorylation, although MKP1 phosphorylation and downstream c-Fos activation were not affected. Interestingly, protease inactive rSap6 was still able to elicit IL-1β release while IL-8 release was nearly absent. Quantification by ELISA assay of IL-8 secreted from OECs showed cells treated with protease inactive rSap6 had a five-fold reduction in secretion (P<0.001) compared with active rSap6 at equivalent concentrations (10 μM) that corresponded to IL-8 secretion produced by addition of 100-fold lower concentration of rSap6 (0.10 μM) (**Figure 3B**). In contrast, OECs treated with rSap6_Δ_ *_RGD_* (10 μM) had impaired MKP1 phosphorylation but similar levels of p-p38 and c-Fos activation as rSap6 (**Figure 3A**). Furthermore, rSap6_Δ_ *_RGD_* induced equivalent secretion of IL-8 as active rSap6 (**Figure 3B**); but OEC treated with rSap6_Δ_ *_RGD_* resulted in substantially less IL-1β release (**Figure 3A**). These results suggested that the RGD integrin-binding domain within Sap6 activated OEC production of IL-1β primarily through MKP1 and c-Fos signaling, while protease activity of Sap6 initiated p38/c-Fos signaling resulting in production primarily of IL-8. Thus, Sap6 appears to have two distinct signaling mechanisms engaging OECs by protease activity or through its RGD integrin-binding domain.

### Both *C. albicans* and Sap6 Induce Expression of PAR2 in OECs

**Figure 4:**
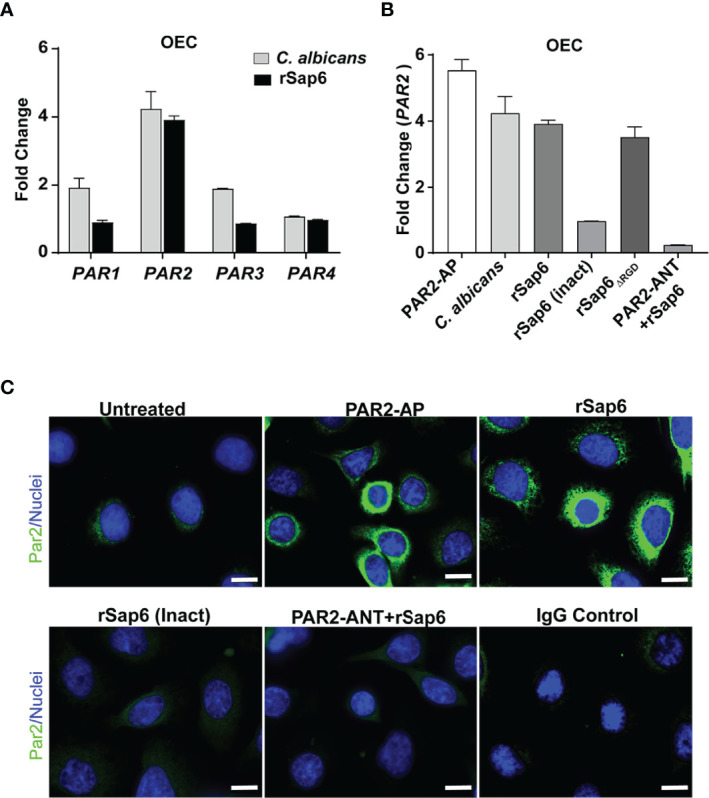
Sap6 primarily induces *PAR2* expression in OECs. **(A)** Changes in gene expression of *PAR1-4* genes in human OECs were calculated by RTqPCR following *C albicans* (5 X 10^6^ cells/ml) or rSap6 (10µM) stimulation. *C albicans* increased expression of OEC *PAR2* (4-fold), *PAR1* (2-fold) and *PAR3* (2-fold) while *PAR4* remained unchanged (grey bars), while rSap6 increased *PAR2* expression levels by 4-fold, and failed to induce expression levels of *PAR1*, *PAR3* or *PAR4* (black bars). GAPDH was used as a housekeeping gene. **(B)** Changes in expression levels of *PAR2* in OECs following 24 h stimulation with *C albicans*, rsap6, rSap6 (Inact) or PAR2-ANT+rSap6 were calculated by RTqPCR. PAR2-AP induced a 5.8-fold increase in OEC *PAR2* expression, similar to that induced by *C albicans* cells, rSap6 or rSap6_*ΔRGD*_. However, rSap6 (Inact) and pre-incubation with a PAR2 antagonist (PAR2-ANT) before addition of rSap6 abolished the increase in *PAR2* expression. **(C)** Cell surface localization of PAR2 (green) was detected using immunofluorescent microscopy following staining of OECs with polyclonal rabbit anti-PAR2 antibody and Alexa Fluor 488-conjugated anti-rabbit antibody. OECs treated with PAR2-AP or rSap6 showed increased level of cell surface localization of PAR2 compared to untreated control cells, while treatment with rSap6 (Inact) or PAR2-ANT did not increase cell surface localization of PAR2. IgG was used as a negative control. Nuclei (blue) were stained with Hoechst. Scale bar shown is 10 µm.

Since we found that Sap6 protease activity is one means of initiating OEC MAPKinase signaling and IL-8 production, we hypothesized that Sap6 protease activity could utilize OECs protease activated receptors (PARs) to activate MAPK/p38 signaling and IL-8 release. Although OECs constitutively express PARs, their surface expression levels are increased following cleavage and downstream activation by binding peptides. To determine which PAR family member expression levels might be induced by Sap6, OECs were infected with *C. albicans* or rSap6 (10 μM) for 24 h and total RNA was extracted from OECs and screened for expression levels of *PAR1*-*4* genes as measured by qPCR. We found that *C. albicans* increased OEC *PAR2* expression levels by 4-fold, and increased *PAR1* and *PAR3* expression by 2-fold, while *PAR4* remained unchanged (**Figure 4A**, grey bars). Incubation of OECs with rSap6 similarly increased *PAR2* expression levels 4-fold, however no changes in expression levels of *PAR1*, *PAR3* or *PAR4* were observed (**Figure 4A** black bars). This result suggested that both *C. albicans* cells and Sap6 are able to activate PAR2 receptors as measured by *PAR2* expression levels, although fungal cells may more modestly activate other PAR receptors.

Next, OECs were pre-incubated with a PAR2 agonist peptide PAR2-AP (SLIGKV-NH_2_) or PAR2 antagonist peptide PAR2-ANT (FSLLRY-NH_2_) as positive and negative controls respectively for PAR2 expression during experiments. PAR2-AP was able to induce a 5.8-fold increase in OEC *PAR2* expression compared to untreated OECs, while either *C. albicans* (5 X 10^6^ cells/ml) or rSap6 (10 μM) induced a 4-fold increase in *PAR2* expression (**Figure 4B**). PAR2 expression levels were dependent upon enzymatic activity of rSap6 since heat-inactivated rSap6 was unable to increase *PAR2* expression. Furthermore, pre-incubation of OECs with a PAR2 antagonist (PAR2-ANT) completely prevented the increase of rSap6-induced *PAR2* expression levels (**Figure 4B**), showing that this effect is specific to PAR2. To confirm that the observed increase in *PAR2* expression levels resulted in a higher PAR2 protein levels on OEC surfaces, we assessed surface localization of PAR2 by immunofluorescence microscopy of cells stained with PAR2 antibody (**Figure 4C**). Indeed, surface PAR2 proteins were highly elevated compared to control cells after OECs were treated with PAR2-AP or rSap6, while treatment of OECs with heat inactivated Sap6 or PAR2-ANT showed little cell surface expression of PAR2. Thus, cell surface expression of PAR2 proteins closely corresponded with *PAR2* gene expression levels.

### Sap6 Induces p38 MAPK Activation While IL-8 Release Is PAR2 Specific in OECs

**Figure 5:**
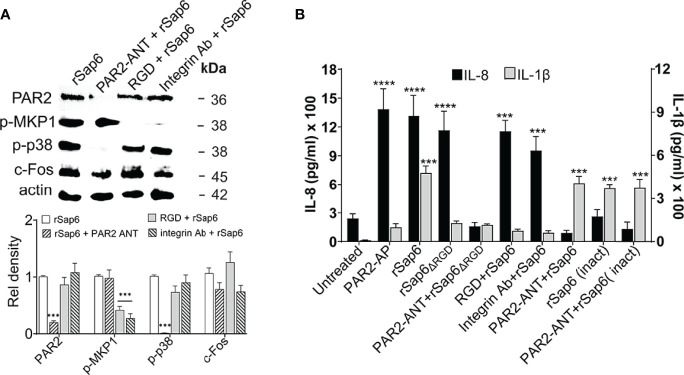
Sap6 RGD domain is required for IL-1β release independent of PAR2 activation while IL-8 release is PAR2 dependent. OECs were treated with rSap6, rSap6*ΔRGD* or rSap6 (Inact) (all at 10µM) or pretreated with PAR2-ANT (100µM), RGD peptide (10µM) or ant-integrin Ab (1:100) for 3 h (for MAPK signaling) or 24 h (for cytokine release in culture supernatants). **(A)** Immunoblotting and densitometry showed that pretreatment with PAR2-ANT significantly reduced rSap6 induced PAR2 levels and p38 phosphorylation, while pretreatment with either RGD peptide or anti-integrin Ab blocked Sap6 induced MKP1 phosphorylation without affecting p38 phosphorylation or PAR2 levels. **(B)** IL-1β (grey bars) and IL-8 (black bars) cytokine levels were measured by ELISA. OECs pretreated with RGD peptide or anti-integrin Ab prior to Sap6 stimulation resulted in an approximately six-fold reduction in IL-1β, similar to that induced by rSap6_*ΔRGD*_, while addition of Sap6 (Inact) or pretreatment with PAR2-ANT resulted in equivalent production of rSap6. OECs pretreated with PAR2-ANT or with Sap6 (Inact) resulted in 10 fold reduction in IL-8 while pretreatment with RGD peptide or anti-integrin Ab prior to Sap6 stimulation did not alter IL-8 release. Data are mean ± SEM of three independent experiments. P values are p ≤ 0.001 (***) and p ≤ 0.00001 (****).

To examine whether PAR2 levels mediates Sap6-induced activation of downstream MAPK and c-Fos signaling in OECs, we first questioned whether PAR2 is required for specific signaling events. OECs were pre-incubated with PAR2 ANT (100 μM) for 24 h and then stimulated with Sap6 (10μM) for 3 h to assess phosphorylation of p38, MKP1 and c-Fos in cell lysates, or for 24 h for IL-8 and IL-1β secretion in OEC supernatants. As expected, there was significant reduction in protein level of PAR2 in OECs pretreated with PAR2-ANT as well as reduced p38 phosphorylation and c-Fos level compared to rSap6 alone, whereas Mkp1 levels were not changed upon PAR2 inhibition (**Figure 5A**). Since MKP1 levels were significantly reduced due to the absence of an RGD motif in rSap6 (**Figure 3A**), we also tested role of integrin for MKP1 activation by rSap6. Pretreatment of OECs with an RGD peptide or anti-Integrin antibody did not affect PAR2 protein levels but significantly reduced MKP1 phosphorylation (**Figure 5A**), again suggesting the presence of a parallel pathway for Sap6 mediated OECs activation by Integrin-specific MAPK signaling. IL-1β release was reduced by approximately four-fold when OECs were pretreated with rSap6_ΔRGD_ (10µM), RGD peptide or anti-integrin Ab (**Figure 5B**). In contrast, Sap6 mediated IL-1β release was not affected by pretreatment of OECS with PAR2-ANT or rSap6 (inact) showing that the PAR2 receptor does not mediate IL-1β production. However, OECs treated with PAR2+ANT followed rSap6, rSap6_ΔRGD_ incubation or rSap6 (Inact) had a six-fold reduction in IL-8 levels compared to PAR2-AP, rSap6 or rSap6_ΔRGD_ (**Figure 5B**), while pretreatment of OECs with either RGD peptide or anti-integrin Ab followed by rSap6 treatment did not change levels of IL-8 release. Thus, PAR2 receptor is required for Sap6 mediated p38 phosphorylation as well as downstream IL-8 release whereas the RGD domain Sap6 plays an important role in Integrin-mediated MKP1 phosphorylation and IL-1β release in OECs.

### Sap6 Degrades OEC Barrier Function and Increases OEC Invasion in a PAR2 Dependent Manner

**Figure 6:**
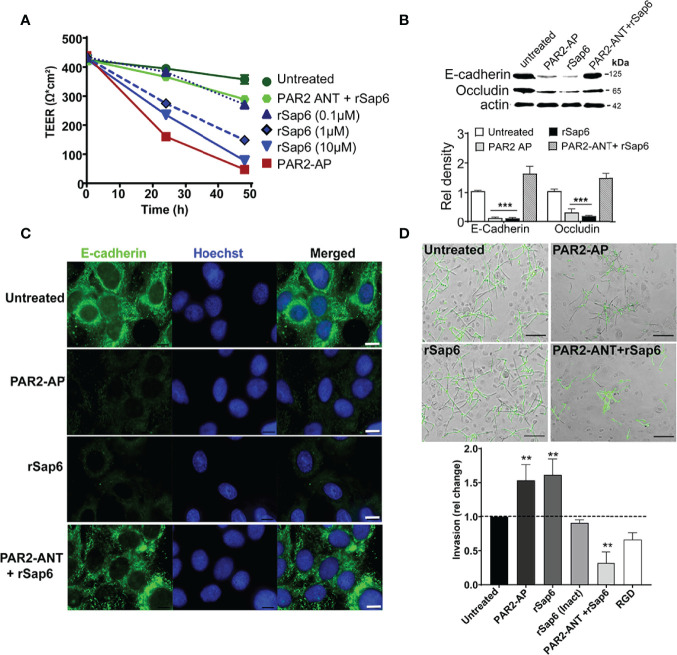
Sap6 mediated PAR2 activation results in decreased epithelial barrier integrity and increases *C. albicans* invasion. **(A)** Changes in TEER value in OECs treated with rSap6 (0.1-10µM), PAR2-ANT+rSap6 and PAR2-AP were calculated over 48 h using an EVOM2 epithelial volt ohmmeter. Measured TEER values were normalized to untreated OECs. There was a dose dependent reduction of TEER value in OECs treated with rSap6 (0.1-10µM) with 10 μM rSap6 causing a 78% reduction in TEER similar to PAR2-AP treated cells. OECs pre-treated with PAR2-ANT before Sap6 treatment did not show any significant change in TEER. **(B)** OEC levels of E-cadherin and occludin proteins were examined by immunoblot and densitometry following 24 h treatment of cells with PAR2-AP, rSap6 or PAR2-ANT+rSap6 using E-cadherin or occludin antibodies. OEC treated with either rSap6 or PAR2-AP showed significant reduced levels of both E-cadherin and occludin compared to OEC control, while pretreatment with PAR2-ANT before Sap6 restored levels of both E-cadherin and occludin to control. Significance was calculated using two-way ANOV compared to untreated OECs. P values are p ≤ 0.01 (**), p ≤ 0.001 (***). **(C)** Cell surface expression of E-cadherin was visualized microscopically following rSap6 or PAR2-AP treatment, then OECs were fixed, permeabilized and stained with E-cadherin (green) and Hoestch (blue). Untreated OECs showed high expression of surface E-cadherin, whereas treatment with rSap6 or PAR2-AP reduced E-cadherin expression that was blocked by pretreatment with PAR2-ANT. Scale bar indicates 10 μm. **(D)** *Candida albicans* cells (1 × 10^5^) were added to OEC monolayers pretreated with PAR2-AP, rSap6, RGD+rSap6 or PAR2-ANT+rSap6. After 4.5 h, epithelial cells were permeabilized and *C albicans* cells were stained with anti-Candida antibody and Alexa Fluor 488 to visualize non-invading cells (green). Invasion was quantitated as percentage fold change in invasion comparing to untreated OECs. OECs treated with rSap6 or PAR2-AP had higher invasion while rSap6 (Inact) did not change invasion levels of *C albicans*. Pre-treatment of OECs with PAR2-ANT or RGD+rSap6 resulted in reduced hyphal invasion. Scale bar indicates 20 µm.

Since *C. albicans* Sap proteases and activation of PAR2 induce loss of tight junction integrity in keratinocytes (36), we questioned whether Sap6 mediated PAR2 activation could modulate *C. albicans* invasion to OECs. Changes in transepithelial resistance (TEER) of OECs treated with rSap6 (0.1 μM- 10 μM) were first measured to identify whether Sap6 alone induced a dose dependent loss of barrier function. Indeed, OECs treated with rSap6 showed a dose dependent reduction in TEER values, with 10 μM Sap6 causing a 78% reduction in TEER values that was similar to that of cells treated with a PAR2-AP alone (**Figure 6A**). However, pretreatment of OECs with PAR2-ANT greatly reduced loss of TEER by Sap6 showing that PAR2 protein is needed for Sap6 mediated disruption of barrier function. We next examined OECs to identify whether loss of TEER corresponded to reduction in levels of junctional proteins E-cadherin and occludin. Western blots of OEC treated with either Sap6 or PAR2-AP showed a significant reduction of levels of both E-cadherin and occludin compared to untreated controls, while blocking PAR2 expression in OECs using PAR2-ANT protected from Sap6 induced loss of these junctional proteins (**Figure 6B**). Immunohistochemistry of OECs stained with E-cadherin-Ab showed that this junctional protein was highly expressed on the surface of untreated cells, but was substantially reduced followed OEC treatment with Sap6 or PAR2-AP (**Figure 6C**). To determine whether this loss of junctional epithelial integrity affected the ability of *C. albicans* to invade OEC cells, OECs were exposed to Sap6 (active or heat-inactivated) or PAR2-AP for 1 h prior of fungal infection. *C. albicans* invasion was assessed by differential staining of hyphae penetrating into OEC after 4 h (**Figure 6D**). OECs treated with rSap6 or PAR2-AP had significantly increased invasion (1.5-fold higher than untreated control cells), while heat-inactivated Sap6 did not change invasion levels of *C. albicans*. However, treatment of OECS with PAR2-ANT before exposure to rSap6 reduced hyphal invasion by 0.5-fold, showing the essential role of PAR2 in mediating OEC invasion and loss of epithelial barrier function.

## Discussion

Oral epithelial cells are a physical barrier and first line of defense preventing fungal colonization and subsequent penetration into connective tissues. Epithelial surface receptors are major components in the host surveillance system against microbial infections that sense microbial danger signals and respond accordingly with downstream cytokine release. Such secreted fungal danger signals include microbial proteases and the toxin candidalysin that come into close contact with epithelial surfaces and subsequently activate both protease-dependent and protease-independent immune responses (20, 37–39). Protease-dependent signaling in response to fungal extracellular proteases has been studied in human airway epithelial cells and is known to cause cytokine release *via* epithelial cell receptors (40, 41). Fungal serine protease Pen c 13 and *Alternaria alternate* aspartyl protease also induced IL-8 expression in human airway epithelial cells or eosinophils by activating PAR1 and PAR2 signaling through the MAPK pathway similar to the action of cysteine protease and serine-like proteases (40, 42, 43). The finding that *C. albicans* secreted Sap6 was able to induce IL-1β, IL-8, IL-6 and TNF-α production in peripheral blood mononuclear cells (38) suggested that similar PAR family members might initiate signaling by similar mechanisms in oral epithelium. Indeed, we discovered that *C. albicans* secreted Sap6 potentiates proinflammatory IL-1β and IL-8 production in primary oral epithelial cells similar to that of *C. albicans* invasive hyphae, and this effect is exerted mostly in PAR2 dependent manner. Surprisingly, recombinant Sap6 induced specific but limited proinflammatory cytokines IL-1β and IL-8 as compared to *C. albicans* hyphae on OECs. This limited cytokine response by OECs upon Sap6 exposure could be due to OECs sensing and executing a narrower cytokine response than immune cells, thus dampening inflammation in the oral environment that has continual exposure to a diverse array of microorganisms.

Our work further strengthens the importance of hyphae secreted proteins in *C. albicans* virulence by assigning new roles for the hyphal specific proteases Sap5 and Sap6. Our data clearly suggested that among these two closely related secreted protease genes, *SAP6* appeared to major player than *SAP5* in activation of p38 MAPK in deletion mutants we tested, suggesting potentially different roles in stimulating OEC responses. In addition to possibly different enzymatic substrate specificities between Sap6 and Sap5, these two proteases also have slightly different RGD motifs (Sap6 contains the classical RGDRGD sequence while Sap5 has an altered RGDKGD motif) that may impact integrin binding with epithelial cells (14). Hence, the divergence of the Sap RGD motif as well as substrate specificity between Sap5 and Sap6 might underly differences in eliciting a MAPK response in OECs. In agreement with work from the Naglik laboratory (3), we found that Saps 1, 2, and 3 that do not contain an RGD motif, also do not activate MKP1/p38 signaling in OECs, suggesting that they lack a major role in the inflammatory response in oral epithelium. However, we found that the RGD motif within Sap6 and integrins induce a parallel pathway for MPK1 phosphorylation and IL-1β release in OECs (**Figures 3**, **5**). Further work is needed to identify the exact nature of this putative integrin binding site in OECs.

Previous attempts to explore the role of PARs in *C. albicans* mediated inflammatory responses *in vitro* using human peripheral blood mononuclear cells (PMBCs) did not find a role for Sap mediated immune responses (38, 39). However, this might be due to differences in expression levels of PARs in PBMCs, since human PBMC monocytes and macrophages mainly express cell surface PAR1 and PAR3 with varying surface levels of PAR2 (44). In contrast, epithelial cells that have significant levels of PAR2 surface expression (19) were activated by microbial and host proteases (45–47). Likewise, airway or esophageal epithelial cells that express surface PAR2 were able phosphorylate p38 MAPK and secrete IL-8 production in response to a PAR2 activating peptide and this signaling was significantly inhibited by PAR2 antagonist peptide (48–51). We found that a PAR2 antagonist reduced OEC activation by Sap6, suggesting a direct role of PAR2. However, it is possible that indirect activation of PAR2 might occur by transactivation of another receptor such as epithelial growth factor receptor (EGFR) that has been shown to be involved in candidalysin mediated immune responses in OECs (20).

On mucosal surfaces, human and microbial proteases are present at varying concentrations. During inflammation, localized higher concentrations of extracellular proteases activate PARs to regulate epithelial permeability and barrier function. *C. albicans* hyphae can invade epithelial cells by degrading E-cadherin (26) and we expected that hyphal secreted proteases including Sap6 might contribute to loss of both adherent and tight junction proteins. In oral or gastro-intestinal epithelial cells, disruption of epithelial tight or adherent junctional proteins accompanies invasion by pathogens and exacerbates inflammation mediated through PARs (22). Activation of PAR2 in oral keratinocytes led to the disruption of tight junctions and increased barrier permeability through the activation of p38 MAPK, leading to release of proinflammatory cytokines (22, 36). *Porphoromonas gingivalis* and its secreted protease gingipain degraded epithelial tight junction protein occludin and adherents-junction protein E-cadherin in oral keratinocytes that was mediated through PAR2 (52). Similarly, activation of PAR2 using Par2 agonist peptides disrupted E-cadherin and compromised the airway epithelial barrier (53). Thus, PAR2 likely serves as a regulatory gateway for preserving epithelial integrity in the presence of various bacteria or fungi.

**Figure 7:**
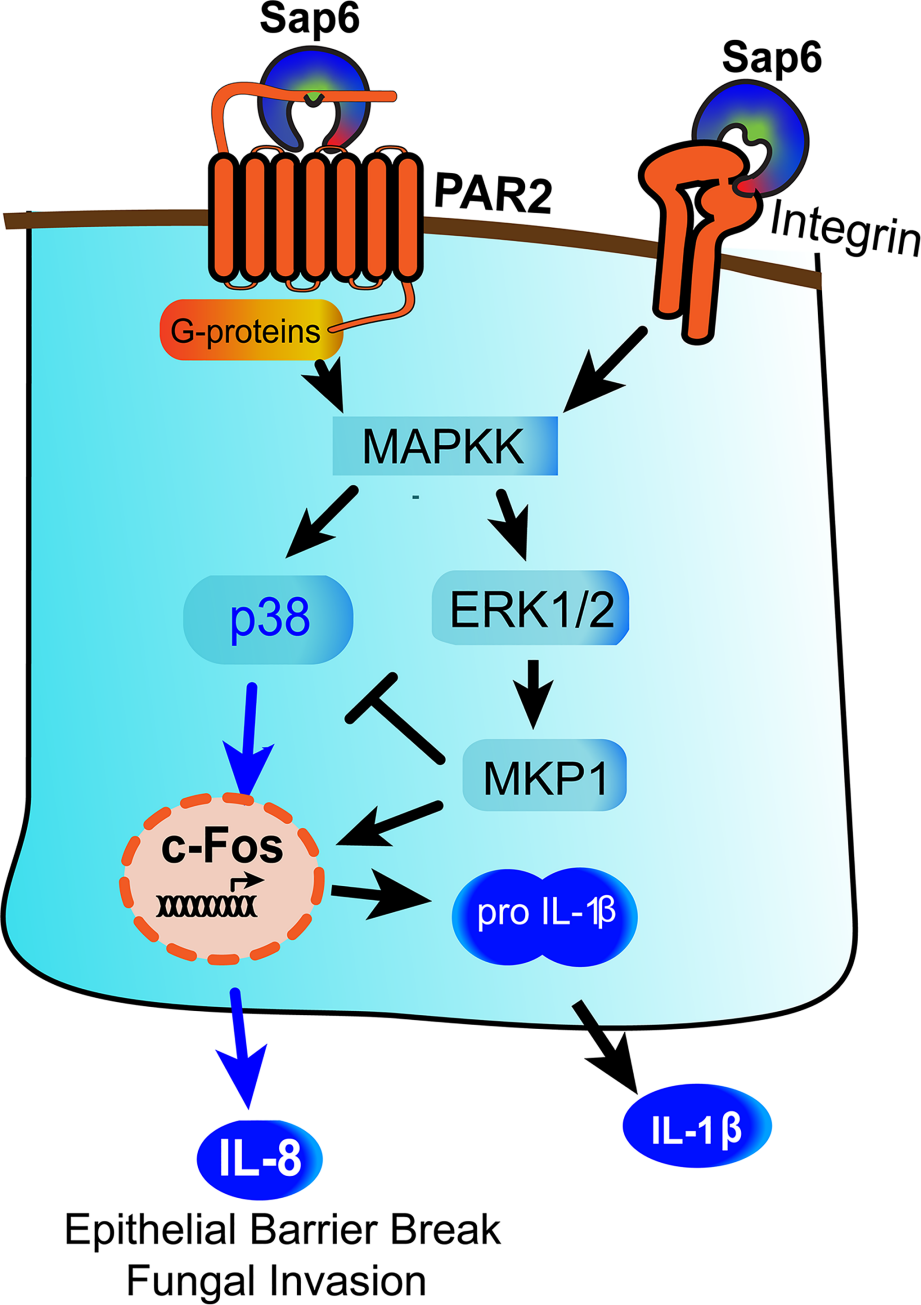
Model for Sap6 mediated oral epithelial cells activation and cytokine release. Sap6 can interact with host cells *via* cell surface receptors including PAR2 or integrins to induce cytokine release through independent pathways. Sap6 protease activity initiates PAR2 signaling by p38 phosphorylation and release of IL-8, while Sap6 RGD domain binds surface integrins to initiate signaling *via* MKP1 to induce IL-1β release. Sap6 mediated PAR2 signaling contributes to loss of epithelial barrier function and increased fungal invasion.

We have identified a novel mechanism by which Sap6 could contribute in *C. albicans* infection through two distinct pathways- first by proteolytic activation of PAR2, and secondly by RGD binding to OEC integrin receptors to initiate an alternative inflammatory response (**Figure 7**). Multiple integrin receptors are expressed on oral or airways epithelial surfaces and are involved in adhesion and invasion of bacterial and fungal pathogens (54, 55). Integrin receptors activate focal adhesion kinases (FAK) that initiate proinflammatory cytokines responses through p38 MAPK signaling and nuclear translocation and activation of NF-κB (56). Along with focal adhesion kinases (FAK), Src family kinases (SFK), or MAPK signaling, microbial protease-cell surface integrin interactions have been associated wiith NLPR3 inflammasome signaling and apoptosis (14, 56–58). Further work to explore this arm of Sap6-integrin interactions and their downstream signaling pathway is needed.

*C. albicans* can take advantage of the disassembly of intercellular junctions to invade deeper into the oral mucosa and disseminate systemically. Direct activation of PAR2 or transactivation of EGFR by Sap6 leading to the disruption of tight junctions and increased epithelial barrier permeability may be an additional invasion mechanism for *C. albicans* virulence. We conclude that PAR2 signaling is a novel and unexplored arm of oral epithelial immunity and that this interplay may function in sensing fungal pathogens.

## Data Availability Statement

The raw data supporting the conclusions of this article will be made available by the authors, without undue reservation.

## Author Contributions

RK and ME conceptualized and designed the experiments. RK performed the experiments and collected the data. RK, IR, and ME analyzed the data and wrote the manuscript. All authors contributed to the article and approved the submitted version.

## Funding

This research was funded by National Institute of Health NIDCR grants R01DE010641 and 1R21DE030245 to ME and 5T32 DE023526-08 to RK.

## Conflict of Interest

The authors declare that the research was conducted in the absence of any commercial or financial relationships that could be construed as a potential conflict of interest.

## Publisher’s Note

All claims expressed in this article are solely those of the authors and do not necessarily represent those of their affiliated organizations, or those of the publisher, the editors and the reviewers. Any product that may be evaluated in this article, or claim that may be made by its manufacturer, is not guaranteed or endorsed by the publisher.
