# Supplementary material for: Candida albicans Sap6 Initiates Oral Mucosal Inflammation via the Protease Activated Receptor PAR2
Source: Front Immunol. 2022 Jun 29;13:912748. doi: 10.3389/fimmu.2022.912748 (PMC9277060; doi:10.3389/fimmu.2022.912748)
Supplement: Supplementary file 1 [file Table_1.docx]

| **Strains** | **Genotype** | **Ref** |  |
| --- | --- | --- | --- |
| SC5314 | Prototrophic clinical isolate | (Fonzi and Irwin, 1993) |  |
| SN250 | *Δura3/::imm434::URA3/ura3::imm434 iro1::IRO1/iro1::imm434 his1::hisG/his1::hisG leu2/leu2 arg4/arg4* | (Noble et al., 2010) |  |
| *Δsap1/2/3* | *sap1Δ::hisG/sap1Δ::hisG sap2Δ::hisG/ sap2Δ::hisG sap3Δ::hisG sap3Δ::hisG* | (Kretschmar et al., 2002) |  |
| *∆sap5* | *his1Δ/his1Δ, leu2Δ /leu2Δ, arg4Δ /arg4Δ, URA3/ura3Δ::imm434,*  *IRO1/iro1Δ::imm434,sap5Δ::C.dubliniensisHIS1/orf19.4831Δ::C.maltosaLEU2* | (Noble et al., 2010) |  |
| *∆sap6* | *his1Δ/his1Δ, leu2Δ /leu2Δ, arg4Δ /arg4Δ, URA3/ura3Δ::imm434,*  *IRO1/iro1Δ::imm434,sap6Δ::C.dubliniensisHIS1/orf19.4831Δ::C.maltosaLEU2* | (Noble et al., 2010) |  |
| *SAP5OE* | *∆ura3::imm434/∆ura3::imm434 RPS1/∆rps1::CIp10-SAP5-URA3* | (Kumar et al., 2015) |  |
| *SAP6OE* | *∆ura3::imm434/∆ura3::imm434 RPS1/∆rps1::CIp10-SAP6-URA3* | (Kumar et al., 2015) |  |

**Table 1. *C. albicans* strains used in this study.**
